# Assessing Exercise-Induced Bronchoconstriction in Children; The Need for Testing

**DOI:** 10.3389/fped.2019.00157

**Published:** 2019-04-26

**Authors:** Natasja Lammers, Maaike H. T. van Hoesel, Marije Kamphuis, Marjolein Brusse-Keizer, Job van der Palen, Reina Visser, Boony J. Thio, Jean M. M. Driessen

**Affiliations:** ^1^Department of Pediatrics, Medisch Spectrum Twente, Enschede, Netherlands; ^2^Faculty of Science and Technology, University of Twente, Enschede, Netherlands; ^3^Medical School Twente, Medisch Spectrum Twente, Enschede, Netherlands; ^4^Department of Research Methodology, Measurement and Data Analysis, University of Twente, Enschede, Netherlands; ^5^OCON Sport, Ziekenhuisgroep Twente, Hengelo, Netherlands; ^6^Department of Sportsmedicine, Tjongerschans Hospital, Heerenveen, Netherlands

**Keywords:** asthma, EIB exercise-induced bronchoconstriction, children, disease history, pediatrician

## Abstract

**Objective:** Exercise-induced bronchoconstriction (EIB) is a specific morbidity of childhood asthma and a sign of insufficient disease control. EIB is diagnosed and monitored based on lung function changes after a standardized exercise challenge test (ECT). In daily practice however, EIB is often evaluated with self-reported respiratory symptoms and spirometry. We aimed to study the capacity of pediatricians to predict EIB based on information routinely available during an outpatient clinic visit.

**Methods:** A clinical assessment was performed in 20 asthmatic children (mean age 11.6 years) from the outpatient clinic of the MST hospital from May 2015 to July 2015. During this assessment, video images were made. EIB was measured with a standardized ECT performed in cold, dry air. Twenty pediatricians (mean years of experience 14.4 years) each evaluated five children, providing 100 evaluations, and predicted EIB severity based on their medical history, physical examination, and video images. EIB severity was predicted again after additionally providing baseline spirometry results.

**Results:** Nine children showed no EIB, four showed mild EIB, two showed moderate, and five showed severe EIB. Based on clinical information and spirometry results, pediatricians detected EIB with a sensitivity of 84% (95% CI 72–91%) and a specificity of 24% (95% CI 14–39%).The agreement between predicted EIB severity classifications and the validated classifications after the ECT was slight [Kappa = 0.05 (95% CI 0.00–0.17)]. This agreement still remained slight when baseline spirometry results were provided [Kappa = 0.19 (95% CI 0.06–0.32)].

**Conclusion:** Pediatricians' prediction of EIB occurrence was sensitive, but poorly specific. The prediction of EIB severity was poor. Pediatricians should be aware of this in order to prevent misjudgement of EIB severity and disease control.

## Introduction

Exercise-induced bronchoconstriction (EIB) is defined as an acute narrowing of the airways that occurs as a result of exercise (1). Evaporative water loss during exercise inducing osmolarity and thermal changes of the airway epithelium plays a central role, leading to smooth-muscle contraction (2). EIB is a specific morbidity of childhood asthma and has a great impact on the quality of life, especially on the physical dimensions (3). Clinical symptoms include shortness of breath, chest tightness, wheeze, and cough in association with physical exercise (4). EIB reflects airway inflammation and is a sign of uncontrolled asthma (5, 6). EIB is diagnosed and monitored based on lung function changes after a standardized exercise challenge test (ECT) according to American Thoracic Society and European Respiratory Society (ATS/ERS) protocol (7, 8). However, in daily practice, ECT's are not often performed and pediatricians usually assess EIB based on a medical history, self- or parent-reported, a physical examination, and possibly lung function. This clinical strategy is in line with guidelines from The Global Initiative for Asthma (GINA), which recommends that an ECT should be undertaken if it is otherwise difficult to assess asthma control (6).

However, it is unclear if this approach is sufficient to properly assess EIB in children. Several studies have shown a poor relation between reported exercise-related symptoms in children and EIB as measured with an ECT (9–11), as well as between basic lung function tests and EIB (12–14). All previous studies focused on separate aspects of clinical information and EIB, while in daily practice, pediatricians combine available information to evaluate EIB and thus asthma control. This study aimed to determine the capacity of pediatricians to predict the occurrence and severity of EIB in asthmatic children, based on clinical information routinely available during an outpatient clinic visit.

## Methods

### Study Design and Patients

This study had a cross-sectional design. Children 6–17 years old with pediatrician diagnosed asthma were recruited from the outpatient clinic of the pediatric department of Medisch Spectrum Twente, Enschede, The Netherlands from May 2015 to July 2015. Children with spirometry-induced bronchoconstriction or severe airflow limitation in baseline spirometry, defined as a forced expiratory volume in 1 s (FEV_1_) < 60% of predicted, were not included. None used short- or long-acting bronchodilators for at least 24 h before the exercise challenge test. Children with other pulmonary or cardiac disorders were excluded. A STARD checklist (31) for this study can be found in the Supplementary Material.

### Study Procedure

#### Clinical Assessment With Medical History, Physical Examination, Video Images, and Spirometry

The clinical assessments and ECT's were performed at OCON sport, in Hengelo. This was carried out by two healthcare professionals, one sports-physician, and one pediatric pulmonologist, with both extensive experience in the clinical assessment of asthma and EIB.

**Figure 1:**
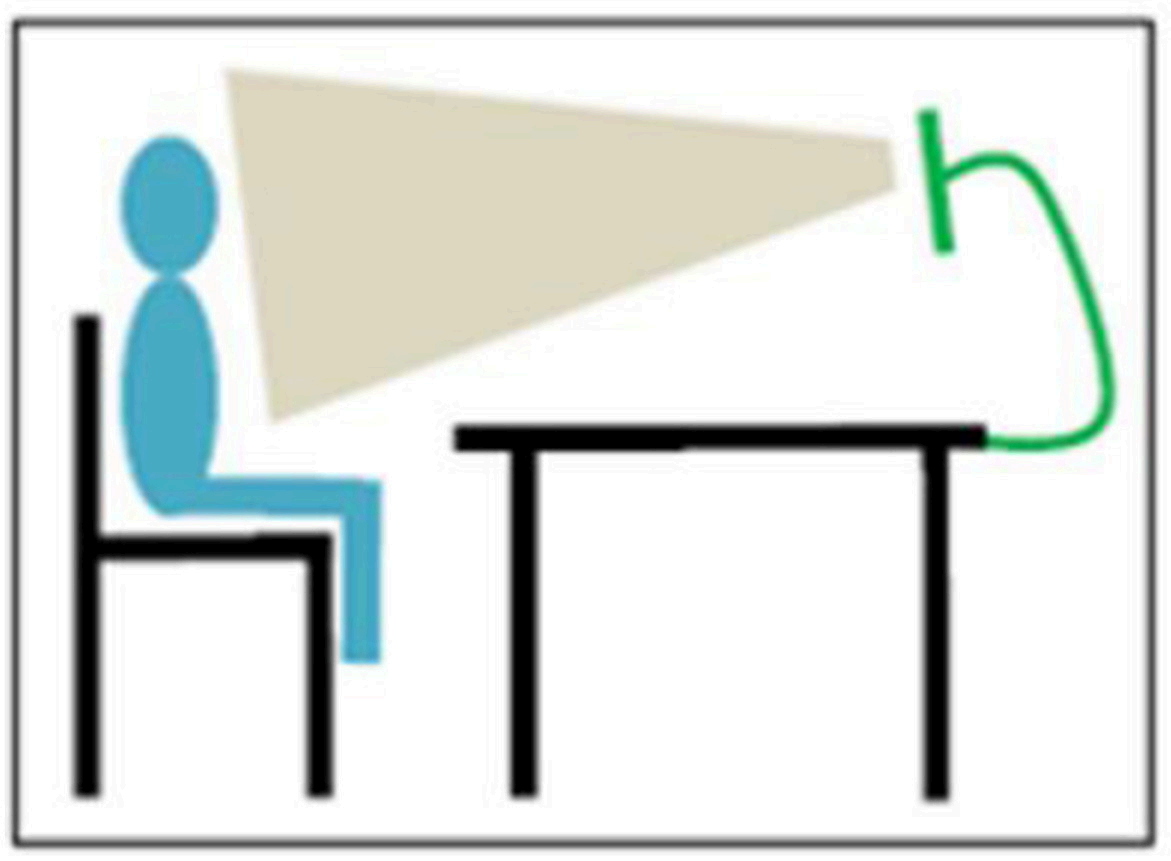
Position of patient and camera while recording.

Before the start of the ECT, a medical history with specific focus on asthma symptoms was obtained (Table 1). Anthropometric measurements were taken (height, weight) and a physical examination (Table 1) including pulmonary auscultation was performed. During this clinical assessment, video images were made in order to provide the pediatricians an overall picture of the children. The video images included sound and were made using an iPad mini attached to a tripod, positioned to film the patients' head and bare chest (Figure 1). After this, baseline spirometry measurements were performed using a MicroLoop® MK 8 hand-held spirometer (ML3535) following standard ATS/ERS protocol (15). Baseline spirometry was measured in duplicate before exercise.

**Table 1:** Elements of medical history and physical examination^a^.

**Medical history**
General asthma symptoms
Exercise-induced symptoms
Nocturnal symptoms
Nasal symptoms
Atopy
Family history positive for asthma
Medication use
**Physical examination**
General impression
Nasal obstruction
Nasal crease (allergic salute)
Vesicular breath sounds
Inspiratory stridor
Expiratory wheezing

a *Part of the clinical assessment before the exercise-challenge test*.

#### Exercise Challenge Test

The ECT's were performed in a climate chamber with cold (10.0–12.0°C), dry air following standard protocol (7). Children aged 8–17 years performed the ECT while running on a treadmill (H/P-Cosmos Quasar 4.0) for 6 min, with an inclination of 10% and with their nose clipped. The treadmill was accelerated until a steady heart rate around 85% of the maximal heart rate (220–age) was achieved in a time period of 2 min. Heartrate was measured with a radiographic ECG-device (Custo cardio 100 BT). Children aged 6 and 7 years performed the ECT on a jumping castle (16), also for a duration of 6 min. Post-exercise spirometry was performed in duplo at 1, 3, 6, 9, 12, and 15 min after the ECT. After post-exercise spirometry, children inhaled 200 μg salbutamol, and 5 min later reversibility was measured. Children with a fall of ≥10% in FEV_1_ post-exercise were considered to have EIB. EIB severity was classified as: 1. No EIB; 2. Mild EIB; 3. Moderate EIB; 4. Severe EIB (Table 2), as suggested by Anderson et al. (17).

**Table 2:** Classification of EIB severity^a^.

**Degree of EIB severity**	**Maximum fall in FEV_**1**_ after exercise**
No EIB	<10%
Mild EIB	>10% but <25%
Moderate EIB	>25% but <50%
Severe EIB	>50% for steroid-naïve patients >30% for steroid-treated patients

a *EIB severity classification, adopted from Anderson et al. (16)*.

The ECT results were interpreted by the above mentioned healthcare professionals.

#### Prediction of EIB by Pediatricians

Twenty pediatricians from three different teaching hospitals (Medisch Spectrum Twente, Isala Klinieken Zwolle, ZGT Almelo/Hengelo) participated in this study. Their average years of experience was 14.4 years (SD 9.8) and two pediatrician were subspecialized as pediatrician-pulmonologist. Each pediatrician independently evaluated five children that were randomly assigned to him or her, providing 100 evaluations in total. The evaluation procedure consisted of two steps.

First, occurrence and severity of EIB (Table 2) was predicted based on the information from the participants' medical history, anthropometric measurements, physical examination and video images. Second, additional information was provided to them in the form of spirometry results [flow-volume curve, FVC (forced vital capacity), FEV_1_, PEF (peak expiratory flow)], after which the pediatricians again predicted occurrence and severity of EIB.

### Statistical Analyses

Results were expressed as mean values ± standard deviation (SD) for the normally distributed continuous data and as median ± interquartile range (IQR) for not-normally distribute data. For nominal or ordinal data, numbers with corresponding percentages were used. The maximum fall in FEV_1_ as a percentage of predicted was calculated and used for statistical analyses.

Sensitivity was calculated as the proportion of children with EIB, diagnosed with an ECT as reference standard, who were given an EIB diagnosis by the pediatricians based on the provided clinical information and spirometry results. Specificity was calculated as the proportion of children without EIB, who were not given an EIB diagnosis by the pediatricians.

The 95% confidence intervals (CI) for sensitivity and specificity were calculated using Episheet (18).

To assess the degree of concordance between the prediction of EIB severity by the pediatricians and the validated classification of EIB based on the ECT, a linear weighted Cohen's Kappa was calculated. Cohen's Kappa values were classified as: <0 = poor; 0–0.2 = slight; 0.2–0.4 = fair; 0.4–0.6 = moderate; 0.6–0.8 = substantial; 0.8–1.0 = almost perfect (19). To assess the difference between two correlated proportions, a McNemar test was used.

For these analysis all 100 evaluations were included, acknowledging the fact that each child was present multiple times in the dataset, albeit assessed by different pediatricians.

A two-sided *p* < 0.01 was considered statistically significant. Data analyses were performed with SPSS® Statistics, version 22.0, and www.vassarstats.net/kappa.html was used to carry out the weighted Cohen's Kappa analyses.

### Ethical Considerations

This study was approved by the Medical Ethics Review Board Twente. All children and parents/guardians received written patient information and provided written informed consent before participating in the study.

## Results

Of 24 children with usable consultation videos, three children had spirometry-induced bronchoconstriction, and one child used salbutamol shortly before the ECT. Twenty children completed the protocol and were included for statistical analyses. Twenty pediatricians independently assessed five children, providing a total of 100 assessments.

### Characteristics of the Study Population

Baseline characteristics of the study sample [10 boys (50.0%)] are shown in Table 3. The mean age of our study group was 11.6 years (SD 3.4). The mean baseline FEV_1_ was 92.7% of predicted (SD 13.9), with a median fall in FEV_1_ after exercise of 15.1% (IQR 1.2–65.1). Nine children showed no EIB, four children showed mild EIB, two children showed moderate and 5 children showed severe EIB (for classification, see Table 2).

**Table 3:** Characteristics of the study sample (*n* = 20)^a^.

**VARIABLES**	
**Sex^b^**	
Female	10 (50.0%)
Male	10 (50.0%)
Age, years	11.6 (3.4)
BMI, kg/m^2^	19.5 (4.6)
Atopy^b^	11 (55.0%)
**Medication use^b^**	
SABA	13 (65%)
LABA	2 (10%)
ICS	10 (50%)
LTRA	6 (30%)
NCS	5 (25%)
Exercise-induced symptoms	8 (40.0%)
FEV_1_ predicted, %	92.7 (13.9)
Fall in ${\text{FEV}}_{1}^{\text{c}}$, %	15.1 (1.2–65.1)
**EIB classification after ECT^b^**	
No EIB (<10%)	9 (45.0%)
Mild EIB (10–25%)	4 (20.0%)
Moderate EIB (25–50%)	2 (10.0)
Severe EIB (>50% or ICS use with >30%)	5 (25.0)
Reversibility^c^, %	18.9 (−11.0–62.3)

a *Values are presented as mean (SD), except when indicated otherwise*.

b *Value is presented as n (%)*.

c *Value is presented as median (IQR). BMI, Body Mass Index (kg/m^2^); SABA, short-acting β2-agonist; LABA, long-acting β2-agonist; ICS, inhaled corticosteroid; LTRA, leukotriene receptor antagonist; NCS, nasal corticosteroid; FEV_1_, Forced Expiratory Volume in 1 s; EIB, exercise-induced bronchoconstriction*.

### Prediction of EIB by Pediatricians

EIB occurrence after the ECT was compared with the predicted occurrence of EIB by pediatricians (Table 4). Based on clinical information and spirometry results, pediatricians detected EIB with a sensitivity of 84% (95% CI 72–91%) and a specificity of 24% (95% CI 14–39%).

**Table 4:** EIB occurrence after the exercise challenge test compared to the predicted occurrence of EIB.

		**EIB after ECT**		
**EIB prediction**		**No**	**Yes**	**Total**
	No	11	9	20
	Yes	34	46	80
	Total	45	55	100

*Prediction by pediatricians, based on medical history, physical examination, pre-exercise video and spirometry results. EIB, exercise-induced bronchoconstriction; ECT, exercise challenge test*.

Table 5 shows an overview of the EIB severity predictions by pediatricians based on the clinical assessment with and without spirometry data and the actual EIB severity after the standardized ECT. EIB was underestimated in the majority of patients with moderate EIB when based on the clinical assessment without spirometry data (14 out of 23 assessments). This improved slightly when spirometry data was added (underestimation in nine out of 23 patients). Without spirometry data, pediatricians underestimated EIB severity in all children with severe EIB. This improved barely when the clinical data was complemented with the spirometry data.

**Table 5:** Overview of predicted and tested EIB severity classifications in participants.

		**EIB severity after ECT**				
		**No EIB**	**Mild EIB**	**ModerateEIB**	**Severe EIB**	**Total**
EIB prediction by pediatricians based on CA	No EIB	11	5	1	4	21
	Mild EIB	22	10	13	6	51
	Moderate EIB	10	5	8	2	25
	Severe EIB	2	0	1	0	3
	Total	45	20	23	12	100
EIB prediction by pediatricians based on CA + spirometry	No EIB	11	5	1	3	20
	Mild EIB	21	11	8	3	43
	Moderate EIB	10	4	11	5	30
	Severe EIB	3	0	3	1	7
	Total	45	20	23	12	100

*EIB, exercise-induced bronchoconstriction; ECT: exercise challenge test. CA, clinical assessment (medical history, physical examination, pre-exercise video)*.

The agreement between the EIB classifications is shown in Table 6. Agreement between the predicted EIB severity classification based on the clinical assessment and the validated classification based on the ECT, was slight [Kappa = 0.05 (95% CI 0.00–0.17)]. This agreement still remained slight when the clinical assessment information was complemented with the spirometry data [Kappa = 0.19 (95% CI 0.06–0.32)].

**Table 6:** Agreement between pediatricians' prediction of EIB severity and EIB severity after an ECT.

	**Kappa (95% CI)**	**Significance**
Clinical assessment * ECT	0.05 (0.00–0.17)	*p* = 0.400
Clinical assessment + spirometry ^*^ ECT	0.19 (0.06–0.32)	*p* = 0.005

*EIB, exercise-induced bronchoconstriction; ECT, exercise challenge test; Clinical assessment, medical history, physical examination, video images*.

Differences between the paired EIB severity classifications, analyzed with the McNemar test, are shown in Table 7. Pediatricians' prediction based on the clinical assessment with and without spirometry data was not significantly different (*p* = 0.181). There was a significant difference between the validated classifications based on the ECT and the predictions based on the clinical assessment, both with and without additional information on pre-exercise pulmonary function (*p* < 0.001).

**Table 7:** Differences between pediatricians' prediction of EIB severity and EIB severity after an ECT.

	**Significance**
Clinical assessment ^*^ clinical assessment + spirometry	*p* = 0.181
Clinical assessment ^*^ ECT	*p* < 0.001
Clinical assessment + spirometry ^*^ ECT	*p* < 0.001

*EIB, exercise-induced bronchoconstriction; ECT, exercise challenge test; Clinical assessment, medical history, physical examination, video images*.

## Discussion

This study aimed to evaluate the capacity of pediatricians to predict the occurrence and severity of EIB based on information routinely available during an outpatient clinic visit. In 100 evaluations, the sensitivity of a pediatricians' predicted diagnosis of EIB was 84%, compared to a specificity of 24%.

The prediction of EIB severity based on a clinical assessment including a medical history, physical examination, and video images of the assessment was poor, with an underestimation of EIB severity in children with moderate and severe EIB. This prediction remained poor when pediatricians were informed about pre-exercise pulmonary function.

To our knowledge, this is the first study that focused on the capacity of pediatricians to predict EIB based on merged data, rather than focusing on separate aspects, available during a routine clinical visit. Our results are in line with previous research that found that clinical information alone is unreliable to predict EIB, leading to both over- and underestimation.

Seear et al. (9) concluded that a respiratory history was unreliable, as it led to the overdiagnosis of EIB in schoolchildren. In a study by de Baets et al. (10) exercise-induced respiratory symptoms after a free running test had a poor positive predictive value for EIB in school-age children not previously diagnosed with asthma. Linna (14) however, was able to predict EIB severity in asthmatic children based on the frequency of general asthma symptoms, including exercise-related symptoms. This is in contrast with other studies that could not identify a relation between EIB and general asthma symptoms assessed with questionnaires, such as the ACT (Asthma Control Test) (20–22) and ACQ (Asthma Control Questionnaire) (23). Moreover, Rietveld et al. (11) did not observe a relation between self-reported dyspnea symptoms and lung function decline during a histamine-induced bronchoprovocation test in their cohort of asthmatic children.

Four studies found no relationship between pre-exercise spirometry values and EIB in children (12–14, 24). Linna (14) however, did find that EIB was related to the concavity of the pre-exercise flow volume curve. Holt et al. stated that spirometry is only meaningful during exacerbations where it influences patients' diagnosis and treatment (25), and not in periods without symptoms due to a low sensitivity.

Studies focusing on athletes with EIB are in line with our results. Hallstrand et al. (26) assessed the accuracy of a medical history and physical examination to diagnose EIB in adolescent athletes and found that half of the EIB diagnoses would have been missed without a standardized ECT. Another study focusing on athletes (27) found that the use of self-reported symptoms for the diagnosis of EIB resulted in both false positive and false negative results.

The unreliability of only using symptoms for the assessment of asthma control was demonstrated by Shefer et al. (28), who found a discordance between children's assessment of general asthma control and the opinion of their pediatricians, with more than 40% of the pediatricians indicating asthma as controlled based on symptom assessment when children indicated it was not. Overestimation of asthma control can lead to undertreatment and creates a potential risk for patients. Several studies have shown that poor asthma control is not only associated with an increased risk of exacerbations, a lower quality of life and increased health-care costs (29), but also with obesity and learning disabilities (30).

A major strength of this study is the standardized exercise challenge tests that were performed in a climate chamber with cold and dry air, following standard protocol. Young children (6–7 years old) performed the test on a jumping castle, a method that has previously been validated by members of our study group (16).

The main limitation of our study is that pediatricians predicted occurrence and severity of EIB based on clinical information not personally obtained. This information was obtained by the investigators of this study during the clinical assessments and ECT's. The pediatricians received this information afterwards and each assessed five children based on the provided information. We complemented this information with video images of the children so that the pediatricians could form a general impression of the children. This study setting is however not a perfect simulation of a real-life setting, and therefore could have led to a less accurate prediction of EIB by the pediatricians.

Another limitation of our study is the inflated sample size: 20 pediatricians each assessed five children (from a total study group of 20 children), providing 100 evaluations. We also acknowledge that therefore each child was present multiple times in our dataset, albeit assessed by different pediatricians.

In conclusion, this study shows that the clinical prediction of EIB occurrence by pediatricians is sensitive, but poorly specific. Furthermore, the prediction of EIB severity based on information routinely available during an outpatient clinic visit is poor. Pediatricians should be aware of this unreliability to prevent misjudgement of asthma control by evaluating EIB without an ECT.

## Ethics Statement

This study was approved by the Medical Ethics Review Board Twente. All children and parents/guardians received written patient information and provided written informed consent before participating in the study. This study was registered on the Dutch Trial Registration under number NTR 5534 (www.trialregister.nl).

## Author Contributions

All except MB-K and JvdP contributed to data acquisition. NL, MB-K, and JvdP contributed to the data analysis. All authors contributed equally on the research protocol, the writing and editing of the manuscript.

### Conflict of Interest Statement

The authors declare that the research was conducted in the absence of any commercial or financial relationships that could be construed as a potential conflict of interest.
